# Treatment of Human Babesiosis: Then and Now

**DOI:** 10.3390/pathogens10091120

**Published:** 2021-09-01

**Authors:** Isaline Renard, Choukri Ben Mamoun

**Affiliations:** Department of Internal Medicine, Section of Infectious Diseases, Yale School of Medicine, New Haven, CT 06520, USA; isaline.renard@yale.edu

**Keywords:** babesiosis, *Babesia microti*, *Babesia duncani*, parasite, therapy, atovaquone, endochin-like quinolones (ELQs)

## Abstract

Babesiosis is an emerging tick-borne disease caused by apicomplexan parasites of the genus *Babesia*. With its increasing incidence worldwide and the risk of human-to-human transmission through blood transfusion, babesiosis is becoming a rising public health concern. The current arsenal for the treatment of human babesiosis is limited and consists of combinations of atovaquone and azithromycin or clindamycin and quinine. These combination therapies were not designed based on biological criteria unique to *Babesia* parasites, but were rather repurposed based on their well-established efficacy against other apicomplexan parasites. However, these compounds are associated with mild or severe adverse events and a rapid emergence of drug resistance, thus highlighting the need for new therapeutic strategies that are specifically tailored to *Babesia* parasites. Herein, we review ongoing babesiosis therapeutic and management strategies and their limitations, and further review current efforts to develop new, effective, and safer therapies for the treatment of this disease.

## 1. Introduction

Human babesiosis is a rapidly emerging tick-born infectious disease caused by intraerythrocytic parasites of the genus *Babesia*. Of several hundred *Babesia* species identified so far, only a few are known to infect humans. These include *Babesia microti*, *Babesia duncani*, *Babesia divergens* and *divergens*-like species, *Babesia crassa*-like, and *Babesia venatorum* [1]. In the United States, most cases of human babesiosis have been attributed to infection with *B. microti*, but sporadic cases due to infection with *B. duncani* and *B. divergens*-like MO1 have also been reported. In Europe, *B. divergens* used to be the main species responsible for infection in humans. However, recent studies suggest that *B. microti* and *B. venatorum* are now more prevalent than *B. divergens* [2]. In China, human babesiosis is mainly caused by *B. microti* and *B. venatorum*, and in the rest of the world, only a few sporadic cases have been reported and were mostly linked to *B. microti* infection [2]. 

**Figure 1:**
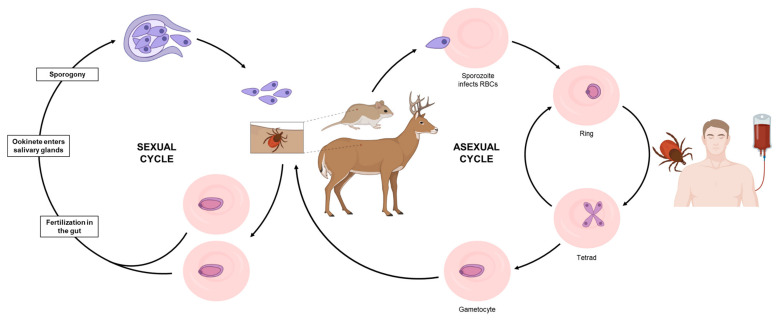
Cycle of transmission of the most common *Babesia* species, *B. microti*. During a blood meal, an infected tick introduces merozoites into the host (mouse or deer, for example). Free merozoites enter red blood cells and undergo asexual replication. While in the blood, some parasites differentiate into male and female gametocytes (not morphologically recognizable by light microscopy). These gametocytes are then taken up by a tick during a blood meal and differentiate into gametes. While in the gut, gametes fuse to form a zygote, that will subsequently undergo meiotic and several mitotic divisions to form sporozoites that are then transmitted to a mammalian host. Humans are typically accidental hosts and become infected through the bite of an infected tick. Human to human transmission is also possible via blood transfusion.

*Babesia* spp. are apicomplexan parasites that infect the host red blood cells and are transmitted to mammals by tick vectors (Figure 1). The species of ticks involved in the transmission of *Babesia* pathogens vary depending on the geographical area and parasite species [1,2]. During the life cycle of *Babesia*, humans are typically accidental hosts, and most infections are linked to a tick route of transmission [1,2]. However, an increasing number of transfusion-transmitted babesiosis cases have been reported in the US over the past 2–3 decades, making *Babesia* infections a major public health concern [1,3,4,5,6]. In 2011, human babesiosis became a nationally notifiable disease in the US [5] and as one of the most common transfusion-transmitted pathogens in the US, *B. microti* was added to the list of significant threats to the blood supply [3,4]. In addition to human-to-human transmission through blood transfusion, several reports have also established the possibility of transplacental transmission from mother to child [1].

In most individuals, babesiosis remains asymptomatic or presents with mild flu-like symptoms [1,2]. However, in more susceptible populations, such as the elderly, asplenic, or immunocompromised individuals, the disease can become severe and even life-threatening, with symptoms such as severe anemia, acute respiratory distress, organ failure, and death [1,2].

In the following sections, we describe the current treatment and management of *Babesia*-infected patients and their limitations. Furthermore, we report on the development and evaluation of novel and highly promising antibabesial therapies.

## 2. Current Treatments against Human Babesiosis

**Figure 2:**
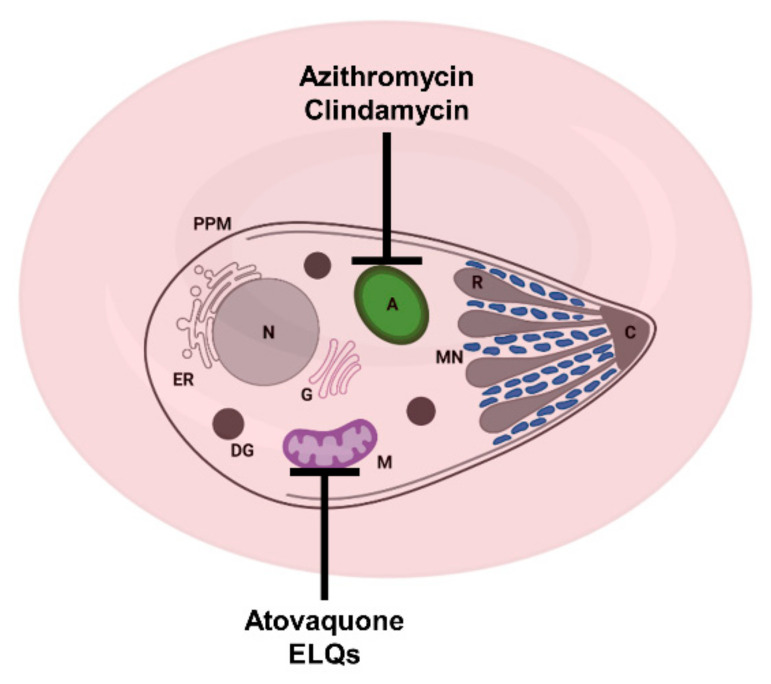
Schematic representation of a *Babesia*-infected red blood cell and sites of action of some approved and experimental drugs. Azithromycin and clindamycin target the apicoplast; atovaquone and ELQs target the mitochondrion. A: apicoplast, C: conoid + polar rings, DG: dense granule, ER: endoplasmic reticulum, G: Golgi apparatus, M: mitochondrion, MN: microneme, PPM: parasite plasma membrane and R: rhoptry.

**Figure 3:**
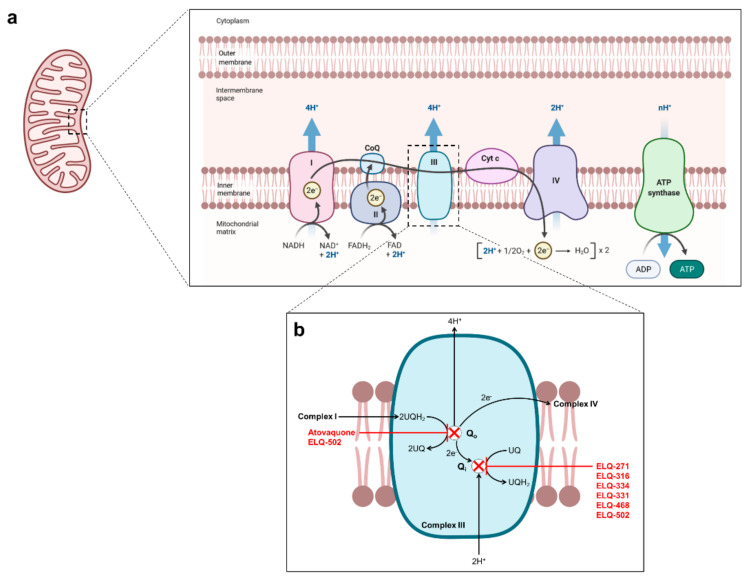
Proposed mechanism of action of atovaquone and endochin-like quinolones in *Babesia* mitochondrion. (**a**) Schematic representation the mitochondrial electron transfer chain. (**b**) Schematic representation of the parasite *bc_1_* complex with proposed mode of action of atovaquone and ELQs.

The current arsenal for the treatment of human babesiosis relies principally on four drugs: atovaquone, azithromycin, clindamycin, and quinine. Atovaquone is used to treat several human diseases, including *Pneumocystis jirovecii* pneumonia [7], toxoplasmosis [8], and malaria (in combination with proguanil (Malarone) [9]. In apicomplexan parasites, atovaquone targets the cytochrome *bc_1_* complex of the mitochondrial electron transport chain (Figure 2 and Figure 3) [10,11,12,13]. Azithromycin is a relatively broad-spectrum antibiotic indicated for the treatment of numerous bacterial infections, such as those caused by *Staphylococcus* spp. [14,15,16] and *Legionella* spp. [17]. The antibiotic is also used for the treatment of toxoplasmosis [12] and, in combination with other drugs, for the treatment of malaria [18]. Azithromycin is a well-characterized protein synthesis inhibitor, which in apicomplexan parasites targets the translation machinery in the apicoplast (Figure 2) [19,20,21]. It is worth noting that azithromycin was found to have a “delayed death” effect, in which parasite division produces viable daughter cells that are subsequently unable to divide in the following cycle [19,21,22]. Clindamycin is another antibiotic commonly used for the treatment of various bacterial infections [23] and repurposed for the treatment of parasitic infections. In combination with quinine, clindamycin is used for the treatment of both malaria and babesiosis [24,25,26]. Several reports have suggested that clindamycin acts in a similar way as azithromycin and targets protein synthesis in the apicoplast (Figure 2) [19,21,22]. Furthermore, selection of clindamycin-resistant *T. gondii* parasites showed cross-resistance to azithromycin, further suggesting a common target [27]. Quinine is a widely used antimalarial agent, typically administered in combination with an antibiotic such as clindamycin or doxycycline [28]. However, the drug is poorly tolerated and, as such, tends to be replaced by alternative drugs with fewer side-effects [28,29]. In malaria parasites, several modes of action for quinine have been proposed. The most commonly reported mechanism of action involves the disruption of hemozoin formation, resulting in accumulation of free ferriprotoporphyrin IX, a by-product of hemoglobin degradation, which is deleterious to parasite growth [30,31,32]. Confocal imaging using fluorescent derivatives of quinine and its structural analogues, quinidine and chloroquine, have shown accumulation of the probes in the digestive vacuole, consistent with the activity of this compound in this organelle [32,33]. Unlike *Plasmodium* parasites, *Babesia* species lack a digestive vacuole, do not degrade hemoglobin, and do not produce hemozoin. Therefore, the mode of action of quinine against *Babesia* parasites is likely to be different from that in *Plasmodium*. Interestingly, fluorescent probes were found to bind to phospholipids and to accumulate in membranous structures, including the parasite plasma membrane, the endoplasmic reticulum, and the mitochondrion, suggesting that quinine may inactivate specific biological functions in these organelles [32,33]. Another proposed hypothesis is that quinine acts as a DNA intercalator [34,35,36]. However, the lack of fluorescence in the nucleus reported by Woodland et al. seem to refute interactions with DNA as a potential mode of action [32,33]. More recently, a study in *P. falciparum* using thermal shift assays suggested that the purine nucleoside phosphorylase (PfPNP) might also be a target of quinine [37].

The severity of babesiosis depends mainly on the host’s immune status, the presence of risk factors and the *Babesia* species responsible for the infection. In symptomatic patients, babesiosis usually manifests with flu-like symptoms such as fever, fatigue, chills, sweats, and headache [38]. For this moderate form of the disease, typically associated with a low parasitemia level (<4%) [26], no hospital admission is required and a 7–10-day treatment course of oral atovaquone + azithromycin (500 mg azithromycin on day 1, followed by 250 mg on subsequent days + 750 mg b.i.d. atovaquone) is recommended [26,38]. Babesiosis typically resolves within seven days from the start of the treatment, but asymptomatic, low level parasitemia may persist for up to one year [26]. Monitoring of persistent parasitemia in immunocompetent individuals following treatment is usually not necessary. However, given the risk of transmission of *Babesia* parasites through blood transfusion, these patients are excluded as blood donors [3]. Immunocompromised individuals are more at risk of developing a severe form of babesiosis, resulting in complications such as acute respiratory distress syndrome, disseminated intravascular coagulation, severe hemolytic anemia, organ failure, splenic rupture, relapse, and death [2,26]. A combination of oral clindamycin + quinine (600 mg + 650 mg, every 8 h) is the standard of care for the treatment of severe babesiosis [26,38]. However, this treatment regimen is frequently associated with serious side effects, such as hearing loss, vertigo, and tinnitus. In some cases, these side effects can be so severe that dose reduction or discontinuation of treatment is required [38]. Recently, it has been demonstrated that a combination of atovaquone + azithromycin is also suitable for the treatment of severe babesiosis, displaying comparable efficacy to clindamycin + quinine with fewer side effects [39]. Although atovaquone + azithromycin is now the preferred course of treatment for severe babesiosis, the standard 7–10-day treatment regimen of oral atovaquone + azithromycin is usually not enough to eliminate *Babesia* infection. Higher doses, longer treatment duration, and in some cases intravenous administration is required to clear the infection [26]. It is also worth noting that the use of immunosuppressive agents such as Rituximab to treat prior illnesses (B cell lymphoid malignancies, rheumatoid arthritis, etc.) may lead to babesiosis relapse and extended persistence of *Babesia* parasites [40,41,42].

One downside of a prolonged treatment regimen and dose escalation is the risk of developing drug resistance. Previous reports have established the emergence of mutations in the cytochrome b (Cytb) of *Babesia* parasites in humans and animal models following treatment with atovaquone [11,42,43]. In 2016, Lemieux et al. examined clinical isolates of relapsing babesiosis and identified a methionine to isoleucine mutation (M134I) in the Q_o_ site (atovaquone-binding site) of the BmCytb [43]. This same mutation was observed in a murine model of *B. microti* infection [11], as well as in other apicomplexan parasites, such as *P. falciparum* and *T. gondii* [43]. Later, Simon et al. reported a Y272C mutation in the BmCytb Q_o_ site in a patient presenting with relapsed *B. microti* infection following an atovaquone + azithromycin treatment course [42]. In both cases, these mutations have been shown to impact the atovaquone-binding domain [44] and appear to be associated with decreased sensitivity to the drug [42,43]. With regard to azithromycin resistance, sequencing of clinical isolates obtained from patients with relapsing babesiosis identified mutations in the ribosomal protein subunit L4 (RPL4) encoded by the apicoplast genome [42,43]. Lemieux et al. identified three substitutions in the RPL4: R86H, R86C and S73L [43]. Simon et al. observed the same R86C mutation in a patient presenting with relapsing babesiosis following atovaquone + azithromycin treatment [42]. Similar mutations associated with azithromycin resistance have been reported in *P. falciparum* [20] and *S. pneumoniae* [45] RPL4. Alternative management strategies for human babesiosis in the case of persistent relapse include the use of different drug combinations such as atovaquone + azithromycin + clindamycin, atovaquone + clindamycin, atovaquone + proguanil, or atovaquone + azithromycin + clindamycin + quinine [26,41,46,47]. The introduction of other drugs such as doxycycline, moxifloxacin, pentamidine, trimethoprim-sulfamethoxazole or artemisinin to treatment regimens with the standard therapies was also reported [40,48]. A recent study in a small cohort of patients suffering from Lyme disease and babesiosis co-infection suggested improvement, and in some cases remission, following one course of disulfiram monotherapy [49]. In patients with high parasitemia (>10%), exchange transfusion is recommended and often results in a rapid reduction of the parasite load [26,50].

Despite clinical evidence that atovaquone, azithromycin, clindamycin and quinine can be used to manage human babesiosis, preclinical evaluation of these drugs in different models of *Babesia* infection has not demonstrated unanimous results with regards to their efficacy. Clindamycin showed only limited activity at a dose of 300 mg/kg (p.o.) in *B. microti*-infected Mongolian jirds [51]. When evaluated in *B. microti*-infected hamster, a course of 150 mg/kg (i.m. or p.o.) of clindamycin resulted in a two-fold decrease in peak parasitemia. Similar results were obtained when clindamycin was administered in combination with quinine [52]. AbouLaila et al. reported a ~three-fold decrease in peak parasitemia following i.p. injection of 500 mg/kg of clindamycin in *B. microti*-infected Balb/c mice [53]. Another study using the same Balb/c model of *B. microti* infection showed that oral administration of clindamycin at 25, 50, and 100 mg/kg did not lead to reduction of parasite burden [54]. Similar results were obtained by Lawres et al. following oral administration of 10 or 50 mg/kg of clindamycin to immunocomprimized mice infected with *B. microti* [11]. The consensus seems to be more apparent in the case of quinine, where most studies report no effect on parasitemia following administration of quinine as a single drug [11,52,54]. Interestingly, a combination of clindamycin + quinine was reported to achieve up to 70% suppression of parasitemia [55] and result in a faster resolution of parasitemia compared to clindamycin alone [52], suggesting a potential synergy between the two drugs. Preclinical investigation of azithromycin efficiency against *Babesia* parasites also yielded inconsistent results. In *B. microti*-infected Balb/c mice, a four-day treatment course with azithromycin at 25, 50, and 100 mg/kg was found to be potent, resulting in 75–96% suppression of parasitemia [54]. In contrast, the evaluation of azithromycin in *B. microti*-infected SCID mice showed no effect on parasitemia at 10 and 50 mg/kg after a seven-day treatment course [11]. Similar results were obtained in *B. microti*-infected hamsters, where 150 mg/kg azithromycin treatment regimen, administered daily for almost two weeks, showed no apparent effect on parasitemia [56]. Out of the four clinically used drugs in the treatment of babesiosis, only atovaquone seems to consistently show high potency against *Babesia* parasites [11,56,57,58,59]. Studies carried out in *B. microti*-infected hamsters and SCID mice reported fast clearance of parasitemia following treatment with atovaquone [11,56]. However, recrudescence due to atovaquone-resistant parasites was observed [11,56]. In *B. microti*-infected hamsters, a combination therapy of atovaquone + azithromycin resulted in rapid clearance of parasitemia without recrudescence [56]. In a lethal model of *B. microti* infection in hamsters, atovaquone monotherapy was found to be superior to a combination of clindamycin + quinine, resulting in low to undetectable parasitemia and extended survival [58]. Potency of atovaquone was also demonstrated in *B. divergens* [59] and *B. duncani* [57] models, with IC_50_ values in the low nanomolar range. In gerbils, although prophylaxis experiments were not successful, a dose of atovaquone as low as 0.5 mg/kg was found to efficiently prevent *B. divergens* infection, so long as daily treatment was maintained several days post-infection [59]. In the case of *B. duncani*, a treatment course of 10 mg/kg atovaquone resulted in a clear reduction of parasitemia and 80% survival using a mouse model of lethal infection [57]. The results derived from the evaluation of atovaquone, azithromycin, clindamycin, and quinine in preclinical models of babesiosis are summarized in Table 1.

While combinations of atovaquone + azithromycin and clindamycin + quinine have been used for more than 20 years for the treatment of human babesiosis [60], the efficacy of these drugs and their primary modes of action in *Babesia* parasites have only recently started to be elucidated. 

**Table 1:** Reported efficacy of atovaquone, azithromycin, clindamycin and quinine in animal models of babesiosis.

Drug	Treatment Regimen	Model	Effect	Ref.
Atovaquone	20 mg/kg (p.o.), 5 d	*B. microti* Balb/c mice	~5.7 × reduction in peak parasitemia.	[61]
	25 mg/kg (p.o.), 4 d	*B. microti* Balb/c mice	77% suppression of parasitemia at DPI 9.	[54]
	50 mg/kg (p.o.), 4 d	*B. microti* Balb/c mice	87% suppression of parasitemia at DPI 9.	[54]
	100 mg/kg (p.o.), 4 d	*B. microti* Balb/c mice	93% suppression of parasitemia at DPI 9.	[54]
	10 mg/kg (p.o.), 7 d	*B. microti* SCID mice	Parasitemia clearance followed by recrudescence by D5-9 post-treatment.	[11]
	10 mg/kg (p.o.), 10 d	*B. microti* SCID mice	Parasitemia clearance followed by recrudescence by D14 post-treatment.	[57]
	10 mg/kg (p.o.), 10 d	*B. duncani* C3H/HeJ mice	Parasitemia clearance followed by recrudescence by D10 post-treatment. 80% survival.	[57]
Azithromycin	25 mg/kg (p.o.), 4 d	*B. microti* Balb/c mice	75% suppression of parasitemia at DPI 9.	[54]
	50 mg/kg (p.o.), 4 d	*B. microti* Balb/c mice	96% suppression of parasitemia at DPI 9.	[54]
	100 mg/kg (p.o.), 4 d	*B. microti* Balb/c mice	95% suppression of parasitemia at DPI 9.	[54]
	10 mg/kg (p.o.), 7 d	*B. microti* SCID mice	No effect.	[11]
	50 mg/kg (p.o.), 7 d	*B. microti* SCID mice	No effect.	[11]
Clindamycin	300 mg/kg (p.o.), 5d	*B. microti* Mongolian jirds	9.4% suppression of parasitemia at DPI 9.	[51]
	150 mg/kg (i.m.), 8d	*B. microti* Golden hamsters	~2× reduction in peak parasitemia.	[52]
	150 mg/kg (p.o.), 8d	*B. microti* Golden hamsters	~2× reduction in peak parasitemia.	[52]
	500 mg/kg (i.p.), 5d	*B. microti* Balb/c mice	~3.2× reduction in peak parasitemia.	[53]
	25 mg/kg (p.o.), 4 d	*B. microti* Balb/c mice	No effect.	[54]
	50 mg/kg (p.o.), 4 d	*B. microti* Balb/c mice	No effect.	[54]
	100 mg/kg (p.o.), 4 d	*B. microti* Balb/c mice	No effect.	[54]
	10 mg/kg (p.o.), 7 d	*B. microti* SCID mice	No effect.	[11]
	50 mg/kg (p.o.), 7 d	*B. microti* SCID mice	No effect.	[11]
Quinine	125 mg/kg (s.c.), 8d	*B. microti* Golden hamsters	No effect.	[52]
	250 mg/kg (p.o.), 8d	*B. microti* Golden hamsters	No effect.	[52]
	25 mg/kg (p.o.), 4 d	*B. microti* Balb/c mice	No effect.	[54]
	50 mg/kg (p.o.), 4 d	*B. microti* Balb/c mice	No effect.	[54]
	100 mg/kg (p.o.), 4 d	*B. microti* Balb/c mice	No effect.	[54]
	10 mg/kg (p.o.), 7 d	*B. microti* SCID mice	No effect.	[11]
	50 mg/kg (p.o.), 7 d	*B. microti* SCID mice	No effect.	[11]
	100 mg/kg (p.o.), 7 d	*B. microti* SCID mice	No effect.	[11]

## 3. In Vitro and In Vivo Models for the Evaluation of Novel Anti-*Babesia* Therapies

Evaluation of the potency of novel drugs for the treatment of human babesiosis has proven challenging due to the absence of a continuous in vitro culture system for *B. microti*, the main causative agent of human babesiosis. A *B. microti* short-term ex vivo system has been used previously for growth inhibition assays [11,62]. However, this culture system is not amenable for high-throughput screening of large libraries of compounds. Despite the current challenges faced in the development of a stable *B. microti* in vitro culture system, this parasite can easily be propagated in rodents, such as mice [63,64,65], hamsters [66], and gerbils [51]. Two very distinct profiles of *B. microti* infection in preclinical models have been observed, depending on the immune status of the host. In immunocompetent animals, such as Balb/c mice, golden hamsters, or gerbils, the parasitemia typically rises within a few days following infection, reaches a peak (40–60% parasitemia), and then resolves on its own [63,64,65]. In immunocompromised animals, such as SCID and rag2D mice, the parasitemia rises and then plateaus at ~50–80% parasitemia [11,57,63]. Immunocompromized mice infected with *B. microti* maintain high parasitemia levels over time but do not succumb to infection [11,57,63]. Although the most commonly used *B. microti* preclinical models (described above) are non-lethal, one research group reported the use of a lethal model of *B. microti* infection in hamsters using the ATCC30222 strain [58,67]. In this model, parasite inoculation results in fulminating disease reaching 90% parasitemia and almost 100% mortality by DPI 12 [58,67]. This model of infection was previously used to evaluate the potency of atovaquone [58].

In vitro culture of *B. divergens*, a species known to infect humans and cattle [1], has been established in mammalian erythrocytes and can be used for the evaluation of potential drug candidates [59,68,69,70,71,72,73]. An in vivo model of *B. divergens* is available in gerbils [74,75] and has been used for the evaluation of potential antibabesial drugs [59,76]. Multiple other rodent species such as rats, mice, hamsters or guinea pigs were tested for the establishment of infection, but none developed parasitemia [74].

The first in-vitro culture system of *B. duncani* in hamster red blood cells was established in 1994 [77]. More recently an adapted protocol of *B. duncani* culture in hamster RBCs was reported using another culture medium [78]. The authors also investigated alternate RBC sources such as mouse, rat, horse or cow. None of these RBCs were able to sustain *B. duncani* growth [78]. In 2018, Abraham et al. reported the first continuous in vitro culture system for *B. duncani* in human erythrocytes [79]. The development of this system allowed for the high-throughput screening of novel derivatives for the treatment of human babesiosis [57]. *B. duncani* can be propagated in hamsters and typically results in fatal infection following the development of pulmonary edema and respiratory distress [80,81]. However, to the best of our knowledge, the *B. duncani* hamster model was not used for the assessment of potential antibabesial drugs. *B. duncani* infection can also be established in mice and is associated with a fatal outcome in specific mouse genetic backgrounds [82,83]. Similar to the hamster model, *B. duncani*-infected mice present with pulmonary edema, leading to respiratory distress and death [83]. Interestingly, it was shown that susceptibility to acute babesiosis following *B. duncani* infection is significantly influenced by the gender and genetic background of the animal [82]. Recently, Chiu et al. presented the first use of a lethal model of *B. duncani* infection in mice for the evaluation of novel promising candidates for the treatment of human babesiosis [57]. The different models of in vitro and in vivo *B. microti*, *B. divergens,* and *B. duncani* available for the evaluation of novel therapeutics are summarized in Table 2.

Overall, there is a wide variety of *Babesia* models available. However, finding complementary systems can prove challenging. Even though *B. microti* accounts for the majority of human babesiosis cases, the absence of a continuous in vitro culture system makes it challenging to use this species for drug discovery purposes. On the other hand, *B. divergens* can be used for in vitro drug screening. However, it’s in vivo model using gerbils may not be widely accessible. Considering this, *B. duncani* appears as the *Babesia* species of choice for drug development. The availability of a stable in vitro culture system in human red blood cells allows for high-throughput screening of large libraries of candidates, offering the possibility to conduct detailed structure–activity relationship studies. Furthermore, the availability of a reproducible model of *B. duncani* lethal infection in immunocompetent mice offers a reasonably affordable option to assess promising drug candidates. 

## 4. Novel Therapies under Investigation for the Treatment of Human Babesiosis 

The recent effort to develop new therapeutics for the treatment of human babesiosis has mostly focused on repurposing known anti-piroplasm agents. A large library of antimalarial drugs, such as artesunate, artemether, dihydroartemisinin, chloroquine, mefloquine, piperaquine, halofantrine, lumefantrine, pyrimethamine, and pyronaridine, has been assessed against *B. microti* but failed to demonstrate much, if any, efficacy against parasite load at the selected dose [51,54,55]. Other antimalarials such as primaquine, pentaquine and robenidine showed potent parasitemia suppression in *B. microti*-infected animals [51,54]. Screening of the Malaria Box, a 400-compound library with known antimalarial activity [84], led to the identification of nine compounds with low micromolar/nanomolar potency (2.1 µM to 160 nM) against *B. divergens* cultured in human erythrocytes [71]. To the best of our knowledge, no further evaluation of the most promising candidates has been reported so far. More recent screenings of the Malaria Box and the Pathogen Box (400 compounds active against neglected diseases) also reported 38 and nine compounds, respectively, with nanomolar potency against *Babesia* species responsible for bovine (*B. bovis* and *B. bigemina*) and equine (*B. caballi*) babesiosis [85,86]. The two most promising compounds identified from the Pathogen Box screening were further assessed in *B. microti*-infected Balb/c mice and showed significant reduction of peak parasitemia [85].

Over the recent years, a large number of drugs, including actinonin [87], atranorin [61], N-acetyl-L-cystein [88], chalcone-4-hydrate [89], trans-chalcone [89], cryptolepine [90], ellagic acid [91], eflornithine [92], fusidic acid [93], gossypol [94], gedunin [95], hydroxyurea [92], luteolin [87,95], nimbolide [95], pepstatin A [96], xanthohumol [94], fluoroquinolone derivatives (enrofloxacin, enoxacin, norfloxacin, ofloxacin, trovafloxacin) [97,98], ciprofloxacin and some of its novel derivatives [53,99], and natural extracts of *Syzygium aromaticum* [100], *Camellia sinensis* [100], *Cinnamomum verum* [101], *Olea europaea,* and *Acacia laeta* [102] have been assessed for antibabesial properties. The in vitro evaluation of these derivatives was mainly carried out against the species responsible for bovine and equine babesiosis and revealed, in most cases, growth inhibition in the micromolar range. In vivo evaluation of these compounds was typically performed in *B. microti*-infected hamsters or Balb/c mice. In most cases, diminution and/or delay in peak parasitemia was observed, but none of the monotherapies displayed high potency against *B. microti* [61,90,91,92,94,97,100,101]. Although some of these compounds could turn out to be promising for veterinary use, they are unlikely to be accepted for clinical use based on their poor selectivity indices. Despite this fact, some of these drugs could be investigated as starting points for structural optimization for the development of novel antibabesial agents.

Out of the multiple derivatives recently reported with potent anti-*Babesia* efficacy, tafenoquine, clofazimine, and endochin-like quinolones are probably the most promising drugs.

Tafenoquine, previously known as WR238605, is an 8-aminoquinoline. In 2018, tafenoquine was approved by FDA for the radical cure of *Plasmodium vivax* infection and for chemoprophylaxis of malaria [103]. Several research groups have investigated the potential of tafenoquine against *Babesia* parasites [55,104,105,106]. In 1997, Marley et al. reported that a twice daily injection of Tafenoquine (i.m.) at 52 mg/kg for four days resulted in 100% parasitemia suppression by day 3 post-drug removal in *B. microti*-infected golden hamsters [55]. Furthermore, a subpassage experiment was carried out to determine whether parasitologic cure was achieved. None of the animals that received blood from tafenoquine-treated hamsters became parasitemic after six weeks post-administration, indicating that treatment resulted in complete cure of *B. microti* infection [55]. More recently, Mordue et al. evaluated tafenoquine in *B. microti*-infected SCID mice [106]. When parasitemia reached ~10%, mice were administered with a single dose of 20 mg/kg of tafenoquine (p.o.). By day 4 post-treatment, parasitemia level was undetectable in tafenoquine-treated animals and remained so until the end of the experiment (day 18 post-treatment). To assess whether the lack of detection of parasites in blood smears was indicative of cure, blood collected from tafenoquine-treated animals at day 18 PT was injected in naive SCID mice. The newly inoculated animals developed detectable parasitemia within one week. Interestingly administration of a single dose of 20 mg/kg tafenoquine (p.o.) resulted in undetectable parasitemia within four days, indicating that the parasites remained sensitive to tafenoquine. In the case where mice were kept beyond day 18 PT, recrudescence was observed day 37 PT. In a separate experiment, *B. microti*-infected mice were treated with a first dose of 25 mg/kg of tafenoquine (p.o.) when parasitemia reached ~20%, followed by a second dose of 12.5 mg/kg of tafenoquine (p.o.) three days later to account for the decrease of plasma concentration. By day 5 after administration of the first dose, parasitemia was below detection level and remained so until day 28 PT. Subpassage of blood collected at day 28 PT in naïve SCID mice resulted in detectable parasitemia with nine days post-inoculation. Overall, although no radical cure was achieved in these experiments, a single oral dose of tafenoquine was found efficient to rapidly reduce parasitemia burden. It is also worth noting that despite the recrudescence observed following treatment, re-emerging parasites did not develop resistance to tafenoquine and remained susceptible to the drug [106]. In 2020, Carvalho et al. investigated tafenoquine in *B. microti*-infected Balb/c mice. Potent inhibition was observed following administration of 10 mg/kg of tafenoquine (three doses on alternate days, p.o.) or of a combination of 10 mg/kg of tafenoquine (three doses on alternate days, p.o.) + 25 mg/kg artesunate (five daily doses, i.p.), starting at day 4 post-infection [104]. In both cases, a ~5.6-fold reduction in peak parasitemia was observed and parasitemia was undetectable from DPI 9 by examination of Giemsa-stained thin-blood smears and remained so until the end of the study (DPI 30). However, except for one animal from the tafenoquine + artesunate treatment group, all mice remained positive for *B. microti* infection by PCR at DPI 27. Interestingly, subpassage of blood collected from tafenoquine-treated mice in a naïve Balb/c mouse resulted in the development of parasitemia, whereas the mouse receiving blood from combination-treated animals remained negative [104]. 

Based on the results described above (summarized in Table 3), tafenoquine could be an interesting drug candidate for further evaluation for the treatment of human babesiosis. With its extended half-life in humans (12–17 days) [106], only a few doses may be required, thus limiting the development of drug resistance. One downside, however, is that tafenoquine causes severe hemolytic anemia in patients with glucose-6-phosphate dehydrogenase (G6PD) deficiency [105,107,108], and as a result its use is contraindicated in such cases. While the exact mechanism of action of tafenoquine in *Babesia* parasites remains unknown, one hypothesis is that the 8-aminoquinoline mediates oxidative stress within the parasite without damaging the host red blood cells of individuals with active G6PD [105]. The latter enzyme plays a key role in the production of NADPH and protects red blood cells from damage by reactive oxygen species (ROS). In the case of G6PD deficiency, NADPH is at a level that is not enough to protect the RBCs from tafenoquine-induced oxidative stress [105].

Clofazimine is an antibiotic used to treat leprosy [111] and drug-resistant tuberculosis [112]. In 2016, Tuvshintulga et al. reported that clofazimine has potent antibabesial effect, following its evaluation in *B. microti*-infected Balb/c mice. A five-day treatment course of 20 mg/kg clofazimine administered either i.p. or p.o. led to suppression of parasitemia by more than 80%, with a slightly superior efficacy when administered orally [113]. Interestingly, although no parasites could be detected by blood smears, blood, heart, spleen, kidney, and liver samples obtained from clofazimine-treated animals tested positive for the presence of *B. microti* ss-rRNA at DPI 40. Consistently, subpassage of blood collected from clofazimine-treated animals in naïve mice resulted in reinfection [113]. It is worth noting that no toxicity was observed in mice during treatment administration. Furthermore, daily administration of 200–300 mg for >30 months for the treatment drug-resistant tuberculosis in humans was well tolerated [114]. More recently, the same research group reported on the efficacy of clofazimine in *B. microti*-infected SCID mice [110]. The continuous administration of clofazimine from DPI 4 to 57 at a daily dose of 20 mg/kg resulted in undetectable parasitemia by examination of blood smears from DPI 14 onward. Parasite DNA could no longer be detected by PCR from DPI 54 until the end of the study (DPI 90), suggesting that this seven-week treatment course is efficient in curing *B. microti* infection [110]. *B. microti*-infected SCID mice treated with 20 mg/kg clofazimine for seven days (DPI4-10) showed no parasitemia by DPI 24. However, recrudescence was observed from DPI 26. Initiation of a second treatment course of clofazimine failed to clear parasitemia, suggesting that the rise of recrudescent parasites was associated with development of clofazimine resistance. Blood samples obtained from two mice that developed recrudescence were sub-passaged in naïve Balb/c mice, which subsequently underwent a five-day clofazimine treatment course. Interestingly, clofazimine successfully impacted the rise of parasitemia in one case, but not in the other [110]. Sequencing analysis established that, unlike atovaquone, clofazimine does not target the cytochrome b of the parasite. As a result, atovaquone-resistant parasites were generated in SCID mice and then propagated in Balb/c mice. A two-week course of 20 mg/kg clofazimine successfully cleared infection in all the mice. However, a relapse was observed in some of the animals, which responded to a second two-week course of a higher dose of clofazimine (40 mg/kg) [110]. Based on these results, clofazimine appears as a promising candidate for the treatment of human babesiosis. Due to the risk of development of drug resistance with a short-term monotherapy, it would be interesting to evaluate the efficacy of clofazimine when combined with a partner drug such as atovaquone. Results derived from the preclinical evaluation of clofazimine are summarized in Table 3.

A novel class of compounds, endochin-like quinolones (ELQs) has recently been reported with high potency against *B. microti* and *B. duncani* [11,57]. Previously reported for their high potency against other apicomplexan parasites such as *Plasmodium* [115,116,117,118,119,120,121,122,123,124,125], *Toxoplasma* [126,127] and *Leishmania* [128], ELQs have been shown to target the cytochrome *bc_1_* complex of the parasites (Figure 3) [117,120,122,128,129,130]. In 2016, Lawres et al. demonstrated potency of ELQ-271 and ELQ-316 in the short-term ex vivo culture system of *B. microti* as well as in the in vivo SCID model of *B. microti* infection. In *B. microti*-infected mice, a seven-day oral administration of 10 mg/kg of ELQ-271 or ELQ-316 resulted in clearance of parasitemia, followed by recrudescence by day 12 post-drug removal [11]. Due to the high crystallinity and low aqueous solubility of this class of compounds, which precludes administration of higher doses, a prodrug of ELQ-316, ELQ-334, was designed by esterification of the carbonyl group present in the quinolone core of the molecule [11,115,116,127]. This strategy led to improved aqueous solubility and increased plasma concentration of the drug following administration of molar equivalents [11,115,116,127]. Administration of ELQ-334 as a monotherapy at 10 mg/kg in *B. microti*-infected mice resulted to slightly extended clearance of parasitemia compared to treatment with ELQ-271 and ELQ-316. However, re-emerging parasitemia was observed by day 16 post-drug removal. In all cases, recrudescence was accompanied by a GCT → GTT mutation in the Q_i_ site of the parasite’s cytochrome *bc_1_* complex, resulting in an Ala to Val substitution at codon 218 [11]. Since monotherapy is not the ideal treatment regimen, a combination of ELQ-334 + atovaquone was evaluated and resulted in complete clearance of parasitemia with no recrudescence following administration of doses as low as 5 + 5 mg/kg [11]. More recently, Chiu et al. reported the screening of a new library of ELQ derivatives against *B. duncani* and identified three potent ELQ prodrugs: ELQ-331 (IC_50_ = 141 ± 22 nM), ELQ- 468 (IC_50_ = 15 ± 1 nM), and ELQ-502 (IC_50_ = 6 ± 2 nM). The previously reported ELQ-316 and its prodrug, ELQ-334 were also assessed against *B. duncani* and showed IC_50_ values of 136 ± 1 nM and 193 ± 66 nM, respectively [57]. 

Further evaluation of the lead candidate, ELQ-502, showed low toxicity in mammalian cells, and thus a highly desirable therapeutic index (>833). ELQ-502 was assessed in *B. duncani*- and *B. microti*-infected mice as a single drug (10 mg/kg) and in combination with atovaquone (10 + 10 mg/kg). Following a 10-day treatment course, both the mono- and the combination therapies resulted in radical cure with no recrudescence, and in the case of *B. duncani*-infected mice, 100% survival [57]. Interestingly, a shorter treatment duration with ELQ-502 alone at 10 mg/kg in *B. microti*-infected mice resulted in recrudescence [109]. Similarly to the results obtained following treatment with ELQ-271, ELQ-316, and ELQ-334, recrudescence following ELQ-502 shorter treatment duration was associated with GCT → GTT mutation in the Q_i_ site of the *BmCytb* [109]. Results obtained from the evaluation of ELQ derivatives are summarized in Table 3.

## 5. Conclusions and Considerations for Future Drug Development

Human babesiosis is an emerging tick-borne disease of rising incidence and a major public health concern. The current therapies for the treatment of human babesiosis are based on drugs already in use against other apicomplexan parasites and tend to be associated with significant adverse effects and/or the development of drug resistance. Moreover, the evaluation of these drugs, namely atovaquone, azithromycin, clindamycin, and quinine, in animal models of babesiosis has raised concerned about their efficacy in achieving parasite elimination. In light of these findings, the need for novel treatments specifically designed to tackle *Babesia* infection becomes apparent. Over the past decades, there has been a growing effort to develop such therapies. Based on their potency, selectivity, and ability to eliminate infection with no recrudescence when combined with atovaquone, endochin-like quinolones (ELQs) appear to be the most promising candidates to advance the treatment of human babesiosis. With regard to the identification of novel molecules with potency against human babesiosis, it could be interesting to establish a standardized protocol for the evaluation of new candidates, in order to facilitate a comparison of results between different research centers. A consensus protocol agreed upon by members of the community and one that follows standard methods for efficacy and safety using established in vitro cell culture assays and in vivo mouse models is warranted.

## Figures and Tables

**Table 2:** Current in vitro and in vivo systems available for *B. microti*, *B. divergens* and *B. duncani* propagation.

*Babesia* Species	In Vitro System	In Vivo Model
*B. microti*	Short-term ex vivo system [11,62]	Mice [63,64,65], hamsters [58,66,67], gerbils [51]
*B. divergens*	Continuous in vitro culture system in human RBCs [69]	Gerbils [74,75]
*B. duncani*	Continuous in vitro culture system in hamster [77,78] and human [79] RBCs	Mice [57,82,83], hamsters [80,81]

**Table 3:** Preclinical evaluation of promising new therapeutics for the treatment of human babesiosis: tafenoquine, clofazimine and endochin-like quinolones (ELQs).

Drug	Treatment Regimen	Model	Effect	Ref.
ELQ-271	10 mg/kg (p.o.), 7 d	*B. microti* SCID mice	Parasitemia clearance followed by recrudescence by D12 post-treatment.	[11]
ELQ-316	10 mg/kg (p.o.), 7 d	*B. microti* SCID mice	Parasitemia clearance followed by recrudescence by D12 post-treatment.	[11]
ELQ-334	10 mg/kg (p.o.), 7 d	*B. microti* SCID mice	Parasitemia clearance followed by recrudescence by D16 post-treatment.	[11]
ELQ-334 + Atovaquone	10 + 10 mg/kg (p.o.), 7 d	*B. microti* SCID mice	Parasitemia clearance throughout experiment.	[11]
ELQ-502	10 mg/kg (p.o.), 5 d	*B. microti* SCID mice	Parasitemia clearance followed by recrudescence by D17 post-treatment.	[109]
	10 mg/kg (p.o.), 10 d	*B. microti* SCID mice	Parasitemia clearance throughout study (DPI 91).	[57]
	10 mg/kg (p.o.), 10 d	*B. duncani* C3H/HeJ mice	Parasitemia clearance throughout study (DPI 91). 100% survival.	[57]
ELQ-502 + Atovaquone	10 + 10 mg/kg (p.o.), 10 d	*B. microti* SCID mice	Parasitemia clearance throughout study (DPI 91).	[109]
	10 + 10 mg/kg (p.o.), 10 d	*B. duncani* C3H/HeJ mice	Parasitemia clearance throughout study (DPI 91). 100% survival	[109]
Tafenoquine	52 mg/kg (i.m.), 4 d (b.i.d.)	*B. microti* Golden hamsters	100% suppression of parasitemia at D3 post-treatment. Reinfection of clean hamster negative.	[55]
	13 mg/kg (i.m.), 4 d (b.i.d.)	*B. microti* Golden hamsters	99% suppression of parasitemia at D3 post-treatment.	[55]
	3.25 mg/kg (i.m.), 4 d (b.i.d.)	*B. microti* Golden hamsters	91% suppression of parasitemia at D3 post-treatment.	[55]
	52 mg/kg (i.m.), 2 d (b.i.d.)	*B. microti* Golden hamsters	99% suppression of parasitemia at D3 post-treatment.	[55]
	20 mg/kg (p.o.), 1 d	*B. microti* SCID mice	Parasitemia clearance followed by recrudescence by D37 post-treatment.	[106]
	25 mg/kg (p.o.), 1 d, + 12.5 mg/kg (p.o.), 1 d (4 d after 1st dose)	*B. microti* SCID mice	Parasitemia clear through D28 post-treatment. Reinfection of “clean” mice positive.	[106]
	10 mg/kg (p.o.), 3 d	*B. microti* Balb/c mice	~5.6× reduction in peak parasitemia.	[104]
Clofazimine	20 mg/kg (p.o.), 52 d	*B. microti* Balb/c mice	Parasitemia clear through DPI 90 (smear + PCR negative).	[110]
	20 mg/kg (p.o.), 7 d	*B. microti* Balb/c mice	Parasitemia clearance followed by recrudescence on DPI 26, unresponsive to a 2nd course of clofazimine 20 mg/kg (p.o.) (14 d).	[110]

## Data Availability

No new data were created. The information presented in this review article are from published reports available in public databases.
